# Laminin Triggers Neutrophil Extracellular Traps (NETs) and Modulates NET Release Induced by *Leishmania amazonensis*

**DOI:** 10.3390/biomedicines10030521

**Published:** 2022-02-23

**Authors:** Gustavo Silva-Oliveira, Leandra Linhares-Lacerda, Thayana R. F. Mattos, Camila Sanches, Tatiana Coelho-Sampaio, Ingo Riederer, Elvira M. Saraiva

**Affiliations:** 1 Departamento de Imunologia, Instituto de Microbiologia Paulo de Góes, Universidade Federal do Rio de Janeiro, Rio de Janeiro 21941-902, Brazil; gustavoimunobioufrj@gmail.com (G.S.-O.); leandralacerda@gmail.com (L.L.-L.); thayanarobertabio@gmail.com (T.R.F.M.); 2 Laboratório de Pesquisas Sobre o Timo, Instituto Oswaldo Cruz, Fiocruz, Rio de Janeiro 21040-902, Brazil; camila.sog@gmail.com (C.S.); riederer@ioc.fiocruz.br (I.R.); 3 National Institute of Science and Technology on Neuroimmunomodulation, Rio de Janeiro 21040-902, Brazil; 4 Laboratório de Biologia da Matriz Extracelular, Instituto de Ciências Biomédicas (ICB), Universidade Federal do Rio de Janeiro, Rio de Janeiro 21941-902, Brazil; tcsampaio@histo.ufrj.br

**Keywords:** laminin, neutrophil extracellular traps (NET), *Leishmania amazonensis*, extracellular matrix

## Abstract

Neutrophils are recruited from the blood and transmigrate through the endothelium to reach tissues, where they are prone to respond through different mechanisms, including the release of neutrophil extracellular traps (NETs). These responses occur in close contact with proteins from the basement membrane and extracellular matrix, where laminins are abundant. Thus, we investigated the interactions between neutrophils and different laminin (LM) isoforms and analyzed the induction of NETs. We showed that neutrophils stimulated with LM isoforms 111, 211, 332, 411, 421, and 511 released NETs. The same occurred when neutrophils interacted with polymerized LMs 111, 411, and 511. LM-induced NETs were partially inhibited by pretreatment of neutrophils with an anti-α6 integrin antibody. Furthermore, NETs triggered by laminins were dependent on elastase and peptidylarginine deiminase (PAD)-4, enzymes that participate in chromatin decondensation. We also found that LMs 411 and LM 511 potentiated the NET release promoted by promastigotes of the protozoan parasite *Leishmania*, and that NETs stimulated by LMs alone display leishmanicidal activity. The ability of LM to induce NET release may have potential implications for the course of inflammation or infection.

## 1. Introduction

In response to infection, neutrophils are recruited from the blood and transmigrate through the endothelium to reach the tissue. Once in tissues, neutrophils are prone to respond to infection through different mechanisms, including the release of neutrophil extracellular traps (NETs). NETs are web-like structures composed of decondensed chromatin associated with proteins from different neutrophil compartments, and they contain and kill microorganisms [1]. Several stimuli trigger the NET extrusion mechanism, which occurs with elastase and myeloperoxidase entry into the nucleus to cleave histones, thereby promoting chromatin decondensation [2,3,4]. Histone citrullination driven by peptidylarginine deiminase (PAD)-4 is also involved in chromatin decondensation during NET formation [5].

To reach the inflammatory site, neutrophils must cross the endothelium and the vascular basement membrane (BM) and NETs have been reported during this transmigration process [6]. Adhesion molecules, including integrins, which are extracellular matrix (ECM) receptors, are also involved in this process [7,8,9]. Laminin is a glycoprotein in the ECM and one of the main components of BMs. Structurally, laminin is formed by the assembly of three polypeptide chains designated α, β, and γ. Mammals possess 5 α, 3 β, and 3 γ chains, and the combinations of these chains form 16 distinct LM isoforms that are expressed in different tissues, where they contribute to the structure of the ECM and modulate cellular functions such as cell adhesion, differentiation, migration, and apoptosis resistance [10,11,12,13].

Laminins are recognized by several integrins that are noncovalently linked αβ heterodimers forming 24 known integrins, 11 of which can bind to LM [14,15,16]. The integrin α6β1 (CD49f/CD29; VLA-6) is expressed on the neutrophil surface and is the most promiscuous receptor, being able to bind to many LM isoforms [17,18,19,20].

Since the effector functions of neutrophils occur in a milieu rich in ECM, where different LM isoforms are present, we decided to study the effect of LM isoforms on NET induction and analyze the LM receptor and signaling pathways involved in this process. Our results showed that the six laminin isoforms tested were able to induce NET release through recognition of the integrin α6 chain. Elastase- and PAD-4-dependent NET release mechanisms were also characterized.

Moreover, neutrophils are among the first cells recruited during the early stages of *Leishmania* protozoan infection [21], and *Leishmania* induces NETs in human neutrophils that trap and kill the parasites [22]. Thus, we tested the ability of LM to modulate NETs triggered by *Leishmania* and examined the leishmanicidal properties of LM-induced NETs. Our data demonstrated increased NET release after neutrophil stimulation by both stimuli and showed that NETs released by LM-stimulated neutrophils are leishmanicidal, indicating that NETs induced by LM may reduce parasite survival.

## 2. Materials and Methods

### 2.1. Laminins

The human LM isoforms 111, 211, 332, 411, 421, and 511 were purchased from BioLamina, Sweden, and used at the indicated concentrations.

To produce polymerized LMs (polyLM), isoforms 111 (Thermo Fisher Scientific, Waltham, MA, USA), 411 and 511 (BioLamina, Sundbyberg, Sweden) were diluted to 50 µg/mL in 20 mM sodium acetate (pH 4) containing 1 mM CaCl_2_. The polymers formed in solution were adsorbed onto glass-bottomed 96-well plates to produce the matrices used for the NET release assays. For the control, the unpolymerized 111, 411, and 511 LMs were diluted to 50 µg/mL in neutral buffer (Tris-HCl 20 mM, pH 7) containing 1 mM CaCl_2_.

### 2.2. Neutrophil Isolation

Peripheral blood collected from healthy donors was used to isolate neutrophils via density gradient centrifugation as described [22]. The procedures involving human biological samples were performed in accordance with the guidelines of the Research Ethics Committee (Hospital Universitário Clementino Fraga Filho, UFRJ, Brazil), approved protocol number: 4261 015400005257.

### 2.3. NET Quantification

Neutrophils (1 × 10^6^) were stimulated with the LM isoforms 111, 211, 332, 411, 421 and 511 with or without *Leishmania* promastigotes (1 × 10^3^) or stimulated with polyLMs 111, 411 and 511. After 90 min, NET release in culture supernatants was quantified using a PicoGreen Kit (Invitrogen, Waltham, MA, USA) and a SpectraMax Paradigm microplate reader (Molecular Devices, San Jose, CA, USA) set at 485/538 nm excitation/emission. PMA (100 nM, Sigma, St. Louis, MO, USA), was used as a positive control.

### 2.4. NET Inhibition Assays

Neutrophils (1 × 10^6^) were treated with elastase inhibitor (IE, 10 μM Merck KGaA, Darmstadt, Germany) or chloroamidine (Cl-A, 12 µM Cayman Chemical, Ann Arbor, MI, USA) for 30 min at 37 °C and 5% CO_2_ and then stimulated with the LM isoforms or PMA for 60 min under the same conditions. NETs were quantified in culture supernatants as described above.

### 2.5. Immunofluorescence

Neutrophils (3 × 10^5^) adhered to 0.001% poly-L-lysine-treated coverslips were stimulated with soluble LM (1 µg/mL, LM suspension group) or directly adhered to either LM- or polyLM-treated coverslips (50 µg/mL, polyLM group) and incubated at 37 °C. After 90 min, the neutrophils were fixed with 4% formaldehyde and blocked against nonspecific binding with 100% AB-positive human serum for 60 min. Cultures were stained with antibodies against pan-LM (1:50 dilution, Sigma), α1 LM chain (100 µg/mL, clone L9393 Sigma), α4 (100 µg/mL, 1:20 dilution, Santa Cruz, Santa Cruz, CA, USA), α5 (1:50 dilution, Millipore, Burlington, MA, USA), anti-human neutrophil elastase (1:500 dilution, Calbiochem) or anti-DNA/histone H1 (1:500 dilution, Millipore) for 1 h at room temperature. Then, goat anti-rabbit or anti-mouse secondary antibodies labeled with Alexa Fluor 488 or 546 (1:300 dilution, Thermo Scientific, Waltham, MA, USA) were added. The slides were mounted in ProLong Gold Antifade Mounting with DAPI (ThermoFisher). Images were obtained with a Zeiss DMi8 confocal microscope.

### 2.6. Integrin Receptor Expression and Inhibition Assays

Neutrophils (1 × 10^6^) were incubated with anti-α6 integrin antibody (GOH3, CD49f-PE, dilution 1:20, BD Pharmingen, San Diego, CA, USA) in 20 µL in RPMI for 15 min at 37 °C, 5% CO_2_, and the expression of α6 integrin was determined by flow cytometry using a FACS Calibur cytometer. The data were analyzed using Summit v.4.3 software. Alternatively, neutrophils were treated with 2 µg/mL of the same anti-α6 integrin antibody and stimulated with LM 111, 411 or 511. An additional 80 µL of RPMI was added, and cultures were incubated for 60 min. NET production was assessed as described above.

### 2.7. Parasite Culture

*L. amazonensis* promastigotes (MHOM/BR/75/Josefa) were grown at 26 °C in Schneider medium (Sigma) supplemented with 10% heat-inactivated fetal calf serum (FCS, Invitrogen), and 40 µg/mL gentamicin (Schering-Plough, Kenilworth, NJ, USA). Promastigotes were obtained after 5 to 6 days of culture (stationary phase), washed three times in PBS (LGC Biotech, Leicester, UK), resuspended in RPMI 1640 medium (Sigma), and used throughout the experiments.

### 2.8. Production of NET-Rich Supernatants

Neutrophils (4 × 10^6^, 200 μL) were incubated with *Leishmania* at the indicated ratios or with 2 µg/mL LM 511 for 4 h at 35 °C, 5% CO_2_. Then, NET-rich supernatants were obtained by centrifugation at 400× *g* for 10 min. Supernatants obtained by stimulation with parasites were again centrifuged at 2760× *g* for 20 min to remove parasites.

### 2.9. Modulation of NET Release

Neutrophils were incubated with or without LM 111, 411, and 511 (1 µg/mL) in solution for 30 min and then further incubated in the presence or absence of *Leishmania* promastigotes (10^3^) for 60 min. NETs in culture supernatants were quantified using PicoGreen. Neutrophils (10^5^) were seeded in 96-well plates and stimulated with 1 µg/mL LM 511 for 30 min. Subsequently, they were stimulated or not with delipidated *E. coli* LPS (10 µg/mL, Sigma-Aldrich) at 37 °C with 5% CO_2_. After 4 h, NET was quantified in the supernatants as above.

### 2.10. Parasite Survival Assay

Promastigotes (1 × 10^6^, 200 μL) were incubated with NET-rich supernatants (0.5 µg/mL) for 2 h at 35 °C, 5% CO_2_, and cell viability was assessed with 4 μM ethidium homodimer-1 (EthD-1, Molecular Probes, Eugene, OR, USA) staining solution for 30 min. Promastigotes killed by 4% formaldehyde served as the positive control. The data were collected with a FACS Calibur flow cytometer and analyzed with FlowJo software.

### 2.11. Statistical Analysis

The data were analyzed with ANOVA or Student t-tests using GraphPad Prism software version 5.00. *p* < 0.05 was considered significant.

## 3. Results

### 3.1. Soluble Laminins Trigger NET Release

**Figure 1:**
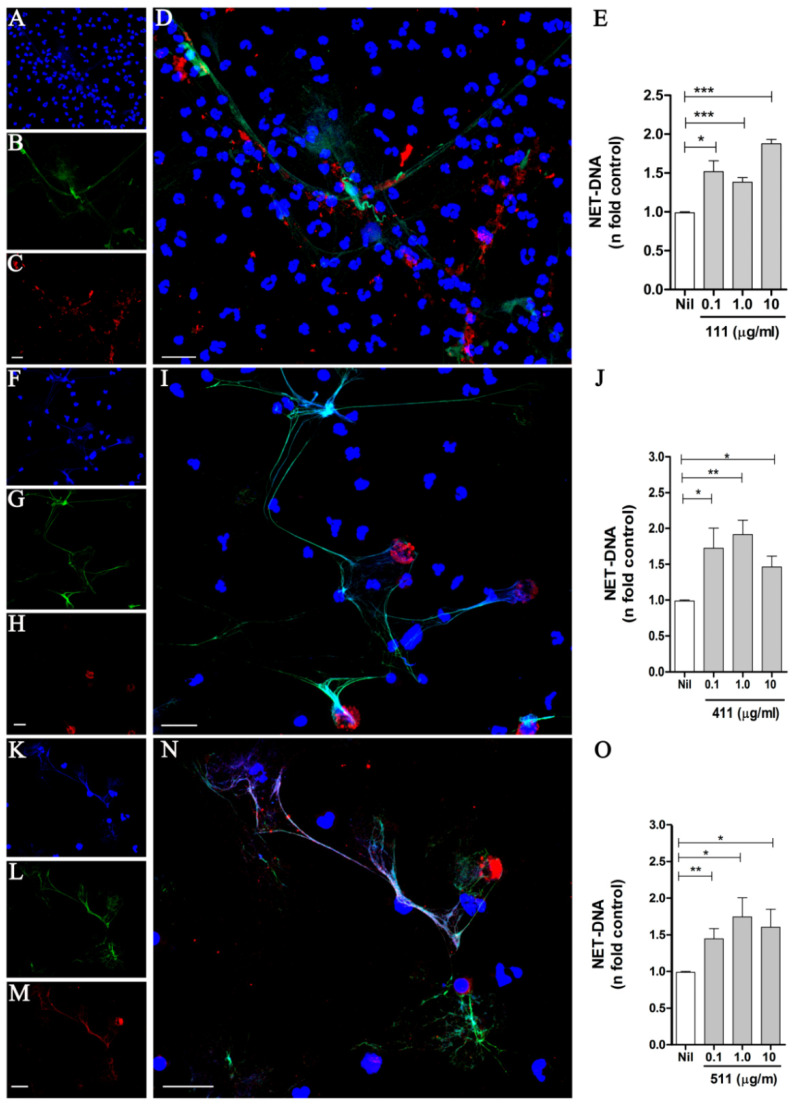
Laminin isoforms 111, 411, and 511 induce NET release. Neutrophils were incubated with the LM isoforms 111 (**A**–**D**), 411 (**F**–**I**), and 511 (**K**–**N**) at 1 µg/mL for 90 min. The cells were then fixed and stained for detection of DNA (with DAPI) (**A**,**F**,**K**), elastase (**B**,**G**,**L**), and LM isoforms 111, 411, and 511 (**C**,**H**,**M**). Enlarged images show merged staining for LMs 111 (**D**), 411 (**I**), and 511 (**N**). Bars: 20 µm. NETs in neutrophil culture supernatants stimulated with LMs 111 (**E**), 411 (**J**), and 511 (**O**) for 90 min were quantified using PicoGreen. The data were normalized according to spontaneous release of DNA (Nil) and are represented as the mean ± SEM of 4 experiments (**E**,**J**,**O**). * *p* < 0.05; ** *p* < 0.004. *** *p* < 0.001.

The endothelial basement membrane (BM) constitutes a barrier for neutrophils to reach the infection site. LMs 411 and 511 are the major isoforms found in vascular BMs and contribute to the neutrophils’ transmigration [23,24,25,26]. To investigate the capacity of LM to induce NETs, human neutrophils were treated with the LM isoforms 111, 411, or 511. After 90 min, cells were fixed and stained with DAPI to detect DNA (Figure 1A,F,K) and with specific monoclonal antibodies to detect elastase (Figure 1B,G,L) and the LM chains α1 (Figure 1C), α4 (Figure 1H), and α5 (Figure 1M). Our results revealed that the LM isoforms were able to induce NETs, which presented the characteristic DNA colocalization with elastase (Figure 1D,I,N). Interestingly, different LM patterns were observed: LM-α1 appeared as small aggregates randomly distributed on the NET scaffold (Figure 1D); on the other hand, LM-α4 appeared in larger, round aggregates without colocalization with the NET meshes (Figure 1I); and LM-α5 colocalized with the NETs (Figure 1N). It has been reported that neutrophils produce LM 411 [27,28], and thus we cannot exclude the detection of LM 411 produced by neutrophils, or alternatively, the recombinant LM added to the cultures could be adsorbed by the NETs and consequently be detected by the antibody.

Next, we quantified NET release by neutrophils stimulated with different concentrations of LM 111, 411, and 511 (Figure 1E,J,O) and observed that all three LM isoforms tested induced NET extrusion at 0.1 to 10 µg/mL. We also showed NET release induced by the LM isoforms 211, 332, and 421 (Figure S1), which presented the characteristic NET morphology (Figure S2). A donor-to-donor variation in the NET extrusion induced by LM was observed, but all isoforms increased DNA release compared with the respective unstimulated control (Figure S3). We chose 1 µg/mL LM to perform the next experiments because all isoforms (except 421) induced NETs at this concentration (Figure 1 and Figure S1). In order to exclude LPS participation in LM-induced NETs, neutrophils were pretreated with Polymyxin B (20 µg/mL) and stimulated with LPS or LM511. We did not observe any effect on the release of NETs generated by LM-511 with or without antibiotic treatment, demonstrating that NETs released are the result of neutrophil contact with the LM isoform and not LPS contamination (Figure S4). Our results demonstrate that the interaction of neutrophils with LMs induces NETs.

### 3.2. Integrin α6 Is Involved in NET Release by Laminins

**Figure 2:**
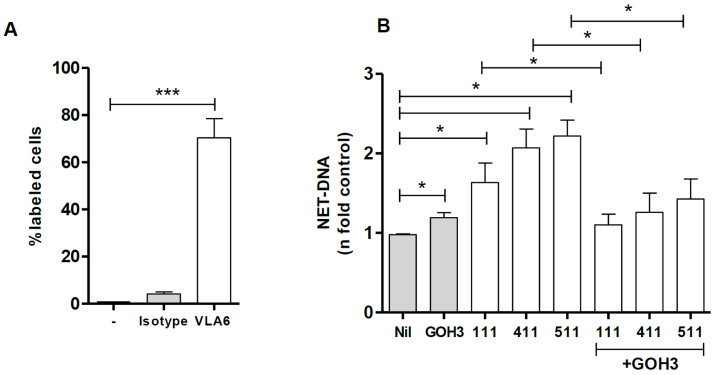
Neutrophils express the α6 integrin chain (CD49f/VLA-6), and NETs are released as a result of recognition of LM isoforms by this integrin. Neutrophils were incubated with anti-α6 chain antibody (GOH3) or the isotype control (2 µg/mL) for 20 min at 4 °C, and CD49f expression was evaluated. The data are shown as the mean ± SEM of the percentage of CD49f+ neutrophils (**A**) in samples from six different donors. Neutrophils were either untreated (Nil) or treated with anti-α6 integrin antibody (GoH3) for 20 min and then incubated with LMs 111, 411, and 511 for 60 min. NETs in culture supernatants were quantified using PicoGreen (**B**). The data were normalized according to spontaneous release of DNA (Nil) from unstimulated neutrophils and are presented as the mean ± SEM of 7 (LM-111), 10 (LM-411) and 8 (LM-511) donors. *** *p* < 0.001; * *p* < 0.05.

It is well-established that neutrophils express integrin α6 (CD49f), which is a laminin receptor [19]. Confirming the literature, we observed that 70% of the neutrophils used in our assays were positive for this receptor (Figure 2A). We next investigated whether inhibition of CD49f by the anti-α6 integrin antibody (GoH3) interfered with the NET extrusion by neutrophils stimulated with LMs 111, 411, or 511. Our data demonstrated that blocking integrin α6 decreased the NETs induced by LM 411, LM 511, and LM 111 by 32%, 35%, and 37%, respectively (Figure 2B). These results demonstrate that NETs induced by LMs 111, 411, and 511 are partially dependent on recognition by the α6 integrin expressed on neutrophils. Donor-to-donor variation in the GoH3 ability to inhibit NET release induced by the LMs is shown in Figure S5.

### 3.3. Signaling Pathways Involved in NET Induction by Laminins

**Figure 3:**
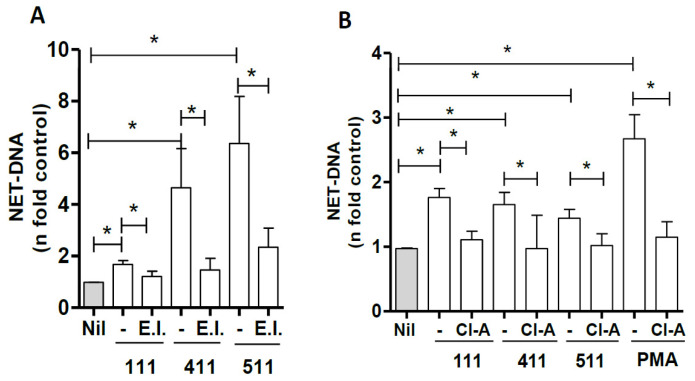
Involvement of Elastase and PAD4 in NET induction by the LM isoforms 111, 411, and 511. Neutrophils were either untreated (Nil) or treated for 30 min with elastase inhibitor (E.I.–A) or PAD-4 inhibitor, chloroamidine (Cl-A–B) and then incubated with LMs 111, 411, and 511 in suspension for 60 min. NETs in culture supernatants were quantified using PicoGreen. The data were normalized according to spontaneous release of DNA from unstimulated neutrophils (Nil) and are presented as the mean ± SEM of 9, 3, and 3 donors for LMs 111, 411, and 511, respectively (**A**); 8, 4, and 4 donors for LMs 111, 411, and 511, respectively (**B**). The data from PMA, which were used as a control, are from 4 donors (**B**) * *p* < 0.05.

To evaluate the signaling pathways involved in NETs induced by LM, we evaluated the role of PAD-4 and elastase. Neutrophils were treated with chloroamidine (12 µM) or elastase inhibitor (10 μM) for 30 min and then incubated with LMs 111, 411, or 511 for 60 min; NET release was then measured in culture supernatants. Our results reveal that the elastase inhibitor decreased the release of NETs stimulated by LMs 111, 411, and 511 by 39%, 68%, and 62.5%, respectively (Figure 3A). Inhibition of PAD-4 with chloroamidine significantly decreased NET release by LMs 111, 411, and 511 (Figure 3B). 

### 3.4. NET Release by Soluble or Plastic-Adsorbed Laminins

**Figure 4:**
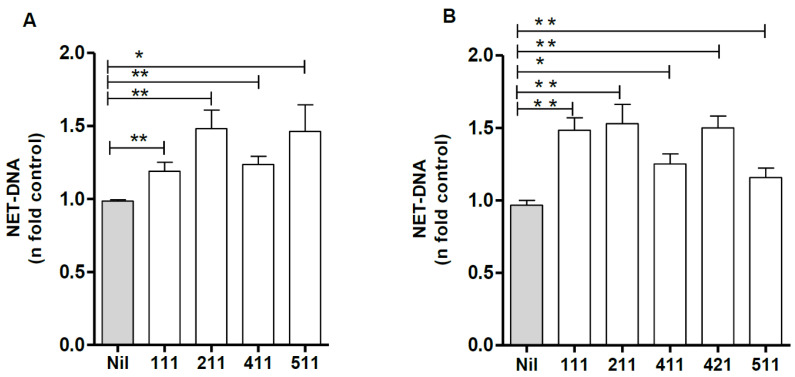
Laminin in solution or adsorbed onto plates induces NET release. Neutrophils were incubated with the indicated LM isoforms in suspension (**A**) or on coated plates (**B**) for 90 min, and NETs were quantified in culture supernatants using PicoGreen. The data were normalized according to spontaneous (Nil) release of DNA and are presented as the mean ± SEM of 7 (**A**) and 3 donors (**B**) for each isoform. * *p* < 0.05; ** *p* < 0.004.

When entering the tissue, neutrophils will adhere and migrate on the existing LMs within the tissue, both in the BM and the interstitial ECM. Moreover, neutrophils can be stimulated by secreted LMs produced by themselves or other cells. To better understand the initial interactions leading to NET formation induced by LM isoforms, we compared the effect of NET release induced by soluble and plastic-adsorbed LMs. Neutrophils were incubated with the indicated isoforms at 1 μg/mL in suspension for 90 min, and DNA was quantified in culture supernatants. Alternatively, NET release was assayed on 10 μg/mL LM-coated plates, and DNA was quantified in culture supernatants. Our data demonstrate that in either form—suspension (Figure 4A) or adsorbed (Figure 4B)—LM induces NET release by neutrophils.

### 3.5. A Polymerized Form of Laminin Induces NET Release

**Figure 5:**
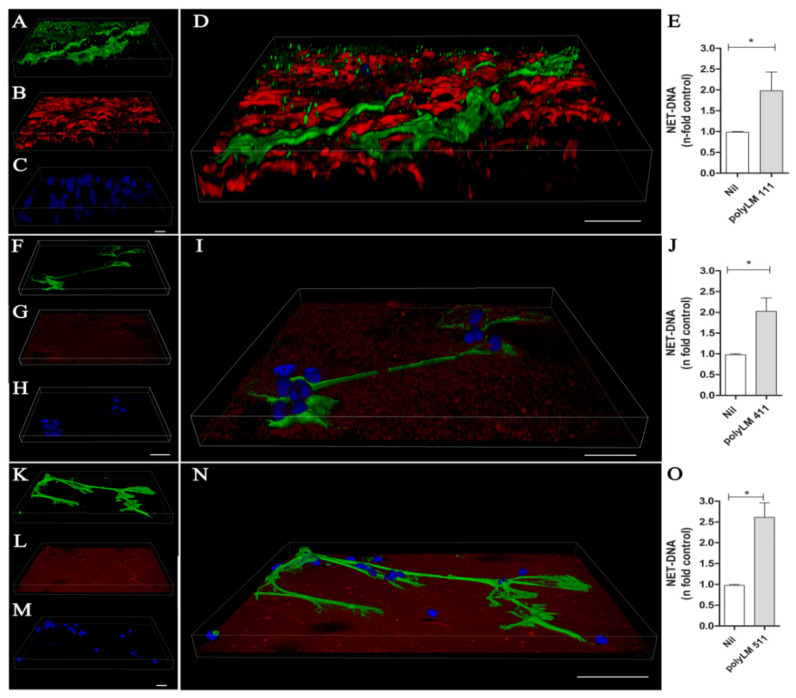
Polymerized laminins (polyLM) 111, 411, and 511 trigger NET release. Neutrophils were incubated on polyLM 111, 411, and 511 (50 μg/mL)-coated plates for 90 min and then fixed and stained for histone (**A**,**F**,**K**); polyLM 111, 411, and 511 (**B**,**G**,**L**); and DNA (**C**,**H**,**M**). Enlarged images show merged staining for polyLM 111 (**D**), 411 (**I**), and 511 (**N**). Bars: 20 µm. NETs in the culture supernatants of neutrophils stimulated with polyLM 111 (**E**), 411 (**J**), and 511 (**O**) for 90 min were quantified using PicoGreen. The data were normalized according to spontaneous release of DNA (Nil) and are represented as the mean ± SEM of 4 experiments (**E**,**J**,**O**). * *p* < 0.05.

Previous studies have shown that acidification induces LM polymerization, giving rise to a hexagonal sheet-like matrix of fractal dimension [29,30]. This matrix is morphologically similar to natural LM matrices assembled by cells at the BM [31], and thus we set out to investigate whether polyLM could distinctly promote NET release. PolyLM formed from LMs 111, 411, and 511 was obtained and adsorbed to a glass surface upon acidification. Neutrophils were then seeded onto the polyLM-coated substrates, incubated for 90 min, fixed, and stained for NET components and laminin isoforms. The results are shown in 3D reconstructions; NETs are labeled with antihistone antibody (green), LM with specific antibodies for each chain (red) and DNA with DAPI (blue) (Figure 5A–N). The polymerization of LM 111 produces a porous matrix, in which neutrophils can penetrate (note that neutrophils are not visible in the merged image) (Figure 5A–D). In addition, we observed NETs from the surface to the bottom of the polymerized layer of LM 111 (Figure S6). NETs released by polyLM 111 were thick, winding, and tightly enmeshed within the matrix structure (Figure S6). In contrast, polymerization of LM 411 produced a shallower polymer, and neutrophils were seen on its surface, together with the NETs generated by their contact with the polyLM 411 (Figure 5F–I). In this case, NETs were straight and lightly touching the coat underneath. On polyLM 511, which formed a compact and tight matrix, neutrophils released NETs morphologically comparable to those seen on polyLM 411 but ending in spread structures resembling neuronal growth cones (Figure 5K–N). Interestingly, these NETs seemed to pull the LM coat (Figure 5L). Notably, the polyLM 111 network itself appeared looser than the polyLM 411 and 511 meshes. NETs were also quantified in supernatants of these cultures (Figure 5E,J,O). The results confirmed that LMs 111, 411, and 511 in their polymerized form were also able to induce NET release.

### 3.6. Laminin Modulates NETs Induced by Leishmania amazonensis

**Figure 6:**
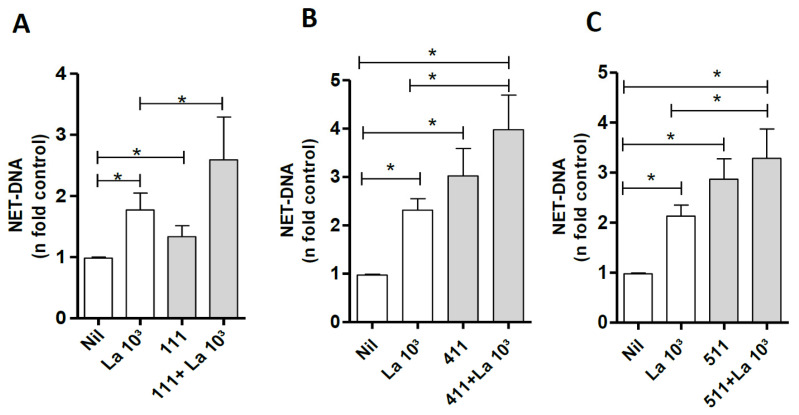
LMs 111, 411, and 511 modulate NETs induced by *Leishmania amazonensis* (La). Neutrophils were incubated with or without LMs 111, 411, and 511 in solution for 30 min and then further incubated in the presence or absence of La (10^3^) for 60 min. NETs in culture supernatants were quantified using PicoGreen. The data were normalized according to spontaneous release of DNA (Nil) and are represented as the mean ± SEM of 6 (**A**), 8 (**B**), and 10 donors (**C**). * *p* < 0.05.

It is known that parasites, including *Leishmania*, can induce NETs [7]. To investigate whether LM can modulate NET induction by *Leishmania,* we incubated neutrophils with LMs 111, 411, and 511 for 30 min and then with the parasite for 60 min. Our data showed 42%, 70%, and 54% increases in NET release when neutrophils were costimulated with *Leishmania* and LMs 111, 411, or 511, respectively, compared with neutrophils stimulated only with *Leishmania* (Figure 6A–C). We also tested if NET release induced by *E. coli* LPS could be modulated by LM 511. Our results showed that NET induction by LPS increased 60% when neutrophils were stimulated by LPS plus LM 511 (Figure S7). 

### 3.7. Leishmania Killing by NET-Rich Supernatants

**Figure 7:**
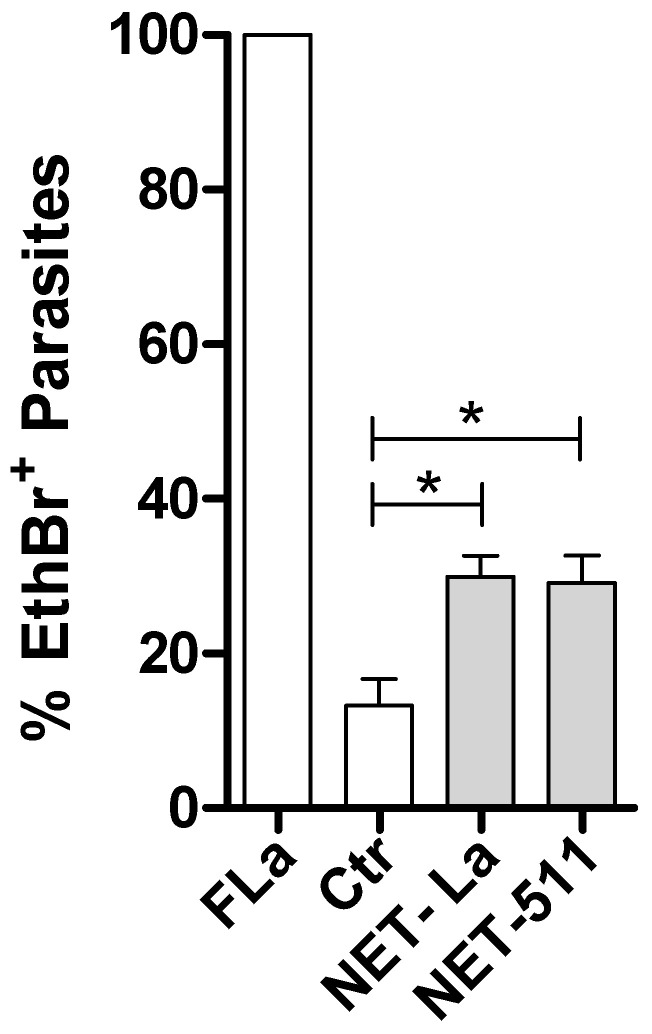
*Leishmania* killing induced by NET-rich supernatants. Promastigotes (1 × 10^6^) were incubated or not for 2 h with NET-rich supernatants obtained after 4 h of stimulation with *L. amazonensis* or LM 511 at 37 °C and 5% CO_2_. Then, parasites were stained with ethidium homodimer-1 (EthD-1) for 30 min at 37 °C and 5% CO_2_ and analyzed via flow cytometry. *Leishmania* fixed with 4% formaldehyde (Fla, dead parasites) served as the positive control (100% of death). The results are shown as the mean  ±  SEM of 2 donors. * *p* < 0.05.

We further evaluated leishmanicidal activity by incubating parasites for 2 h with NET-rich supernatants obtained after neutrophil stimulation with *Leishmania* or LM 511 and evaluated parasite viability (Figure 7). Our results showed that NETs induced by the parasites or by LM 511 induced 2 times more parasite death than that observed in the untreated control. This result indicates that NETs released by neutrophils stimulated with LM 511 possess leishmanicidal activity similar to that of NETs released by neutrophils stimulated with *L. amazonensis* alone.

## 4. Discussion

Upon different stimuli, neutrophils leave the blood vessels, cross the endothelium and the basement membrane, and move to the inflammatory focus through chemotactic, haptotactic, and topotactic stimuli [7,8,9]. Cell–cell and cell–ECM interactions through adhesion molecules, such as integrins, are fundamental for this process [32]. Indeed, neutrophils perform their effector functions immersed within an ECM network [33]. The LM family of heterotrimeric (αβγ) glycoproteins stimulates several biological processes, influencing the function of immune cells, including the increased chemotactic activity of neutrophils, by modulating the expression of their chemotactic receptors [34,35,36]. The BM of vascular endothelium contains LM 411, 421, and 511 isoforms [37,38]; and LM 332 is the major LM in the epithelial BM of the skin [39]. LM 211 is present in the BM of muscle fibers [40], and LM 111 is upregulated and widely expressed during embryogenesis, but exhibits a restricted distribution in adults and is only found in tissues such as the eye, liver, and kidney [41]. Since neutrophils must cross an LM layer in the BM to reach the inflammatory foci, and that LM regulates multiple signaling pathways in leukocytes [34], we thus investigated the capacity of different LMs to stimulate NET release by neutrophils. We showed that all isoforms tested induced NET extrusion. Furthermore, LMs 111, 411, and 511, either in solution or adsorbed to a plastic surface, induced NET release by human neutrophils, and no differences in terms of NET quantity were detected among these two types of LM presentation.

LM is recognized by different receptors, including several integrins, which mediate a diversity of effects in leukocytes [34]. The role of integrins in NET formation has been described for neutrophils stimulated with platelets via the leukocyte integrin Mac-1 (αMβ2; CD11b/CD18) and for CD11b in poxvirus-induced NETs [42,43,44,45,46]. NET release has also been shown to result from binding of the *Yersinia pseudotuberculosis* invasin protein to the β1 integrin chain [44].

The expression of integrin α6β1, the most ubiquitous and specific receptor for LM isoforms, on neutrophils is increased during transendothelial migration [18,19,47]. Here, we demonstrated that the antibody-mediated blockade of α6 integrin significantly decreased NET extrusion induced by the LM 111, 411, and 511 isoforms, implicating this integrin subunit in NET release induced by LM. Nevertheless, the participation of other receptors in LM-induced NET extrusion cannot be excluded. Remarkably, fungal β-glucan in the presence of fibronectin induces NET formation that is dependent on recognition by αMβ2 integrin, also known as complement receptor 3 (CR3), MAC-1, or CD11b/CD18 [34,48].

Various stimuli may induce NET release and activate different signaling pathways [4]. LM-induced NET release is dependent on elastase and PAD-4, because inhibition of these two enzymes decreased NET extrusion induced by LMs 111, 411, and 511.

It has been reported that acidification induces LM self-polymerization into a three-dimensional polymer presenting the overall appearance of natural matrices [29,49]. This polymerized LM (polyLM) has important biological functions, such as promoting regeneration after experimental spinal cord injury, due to inhibition of the inflammatory process and is more efficient in neuritogenesis and neuronal differentiation and in stimulating retinal cell proliferation than non-polymerized LM [30,50]. To better mimic the spatial configuration of LM, we tested NET extrusion induced by polymerized LM. Interestingly, although NET extrusion was observed upon neutrophil interaction with polyLM produced from all three isoforms, the respective NET morphologies were quite different. In polyLM 111, thick NETs were deposited above and between the characteristic spaces formed by the LM polymerization. The thinner polyLM 411 triggered slender NETs that did not penetrate the polymer, and polyLM 511 induced NETs that seemed to pull back the LM and were able to wrap polyLM in the NET contact zone, suggesting a strong interaction.

During *Leishmania* infection, promastigote forms of the parasite are inoculated by the insect vector into a blood pool in the host dermis, allowing contact between the parasites, neutrophils, and ECM. Since our group has been investigating NET release during *Leishmania* infection [22,50,51], we wondered if neutrophils would modulate the NET formation process in response to two stimuli (the parasite and LM). Our results demonstrate that NETs induced by LM 111, 411, and 511 exert an additive effect on NETs induced by *Leishmania*. Furthermore, an additive effect was demonstrated for another proinflammatory stimulus, as LM 511 potentiated LPS-induced NET formation.

It has been demonstrated that NETs are toxic to several microorganisms such as bacteria and fungi, and our group has demonstrated that *L. amazonensis* promastigotes are killed by NETs [22,50]. Pathogens can benefit from the host ECM molecules and use LM as an anchorage point for further invasion [52]. Of particular interest, the interaction of *Leishmania* with LM may influence the homing of the parasite [53]. In this work, we demonstrate that LMs not only stimulate NET release but increase the *Leishmania* capacity to induce NETs and are toxic to the parasites.

Taken together, these data demonstrate that LM isoforms may contribute to the effector mechanism of neutrophils, although further studies are needed to elucidate the impact of these interactions in the course of inflammation or infection.

## Data Availability

The data that support the findings of this study are available from the corresponding author upon reasonable request.

## Supplementary Materials

The following supporting information can be downloaded at: https://www.mdpi.com/article/10.3390/biomedicines10030521/s1, Figure S1: Laminin isoforms 211, 332 and 421 induce NET release; Figure S2: Morphology of Laminin isoform induced NETs; Figure S3: Donor-to-donor variation in NET release induced by the indicated LM isoforms; Figure S4: NET induced by LM is not the result of LPS contamination; Figure S5: Donor-to-donor variation in the NETs released and antibody mediated inhibition of the α6 integrin chain; Figure S6: NETs were entangled in PolyLM 111; Figure S7: LM-511 modulate NETs induced by *E. coli* LPS.
