# Supplementary material for: Laminin Triggers Neutrophil Extracellular Traps (NETs) and Modulates NET Release Induced by Leishmania amazonensis
Source: Biomedicines. 2022 Feb 23;10(3):521. doi: 10.3390/biomedicines10030521 (PMC8945559; doi:10.3390/biomedicines10030521)
Supplement: Supplementary file 1 [file biomedicines-10-00521-s001.zip › biomedicines-1608058-supplementary.pdf]

Laminin triggers neutrophil extracellular traps (NETs) and modulates NET release induced by *Leishmania amazonensis*.

Gustavo Silva-Oliveira[1], Leandra Linhares-Lacerda[1], Thayana R.F. Mattos[1], Camila Sanches[2,3], Tatiana Coelho-Sampaio[4], Ingo Riederer[2,3], Elvira M. Saraiva[1*]

1- Departmento de Imunologia, Instituto de Microbiologia Paulo de Goes, Universidade Federal do Rio de Janeiro, Rio de Janeiro, Brazil;

2- Laboratório de Pesquisas sobre o Timo, Instituto Oswaldo Cruz, Fiocruz, Rio de Janeiro, RJ, Brazil;

3- National Institute of Science and Technology on Neuroimmunomodulation, Rio de Janeiro, Brazil

4- Laboratório de Biologia da Matriz Extracelular, Instituto dee Ciências Biomédicas (ICB), Universidade Federal do Rio de Janeiro, Rio de Janeiro, Brazil.

*** Correspondence:** Elvira Saraiva (esaraiva@micro.ufrj.br).

Supplementary information

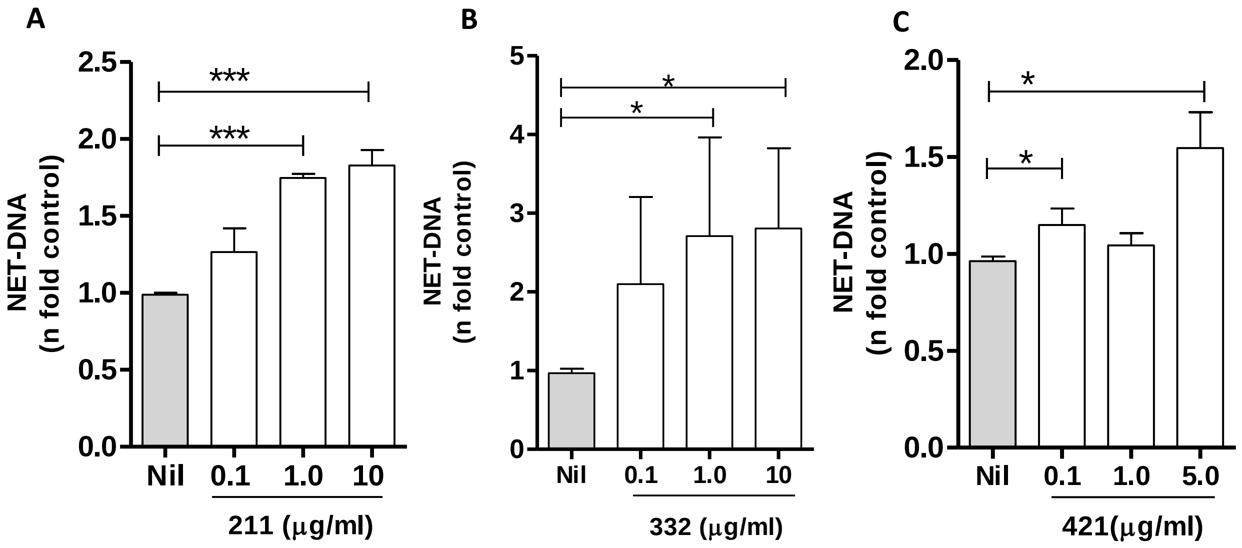

Figure S1. *Laminin isoforms 211, 332 and 421 induce NET release.* Neutrophils were incubated with the LM isoforms 211 (A), 332 (B) and 421 (C) at the indicated concentrations for 90 min. NETs in culture supernatants were quantified using PicoGreen. The data were normalized according to spontaneous release of DNA (Nil) and are presented as the mean ± SEM of 4 (A), 3 (B) and 4 (C) donors. * $p < 0.05$; *** $p \leq 0.001$.

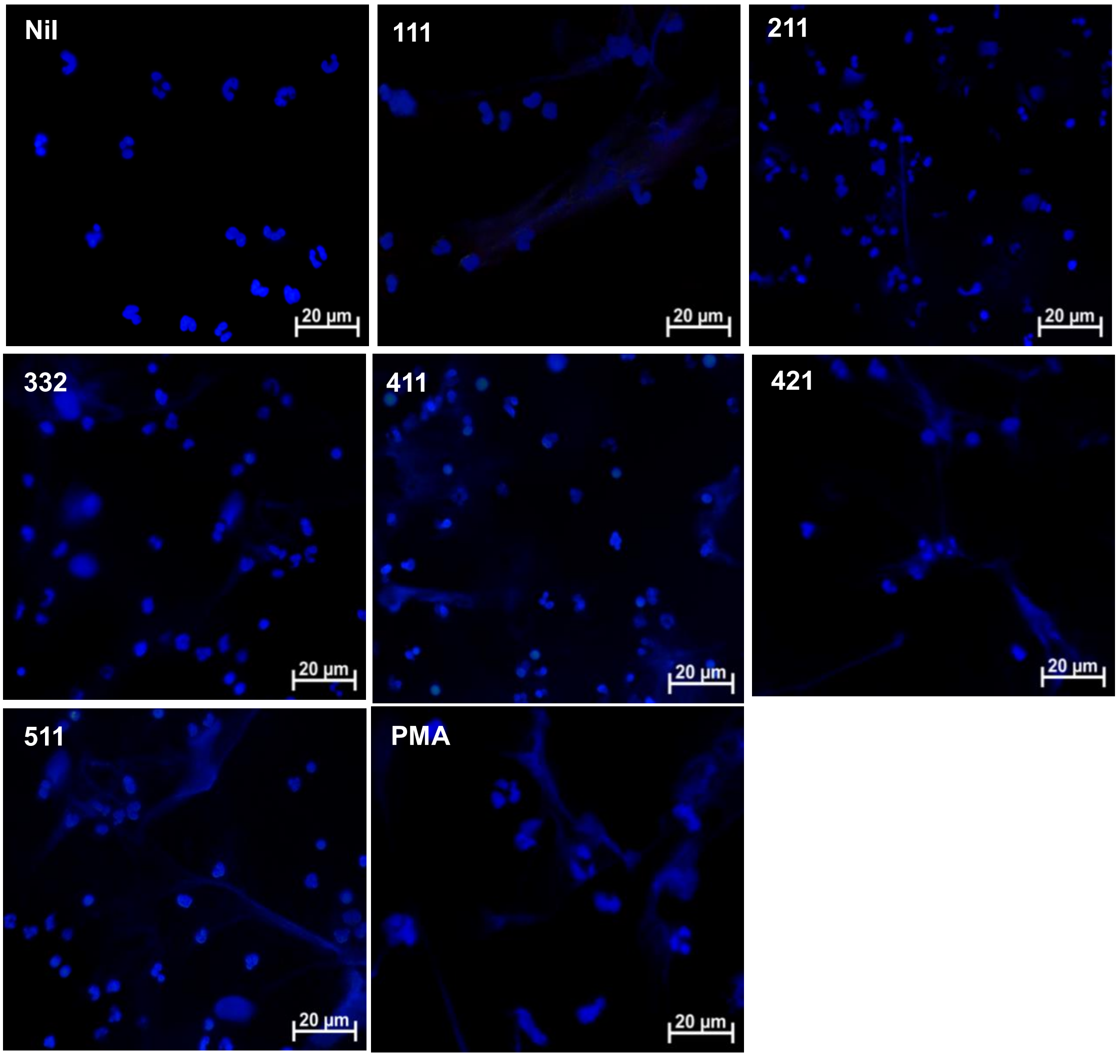

Figure S2. *Morphology of Laminin isoform-induced NETs.* Neutrophils incubated with the indicated LM isoforms (1 µg/ml) and PMA (100 ng/ml) for 90 min, were stained for DNA with Hoechst (1 µg/mL). NETs were visualized using a Carl Zeiss AG Microscope "Axio Imager 2".

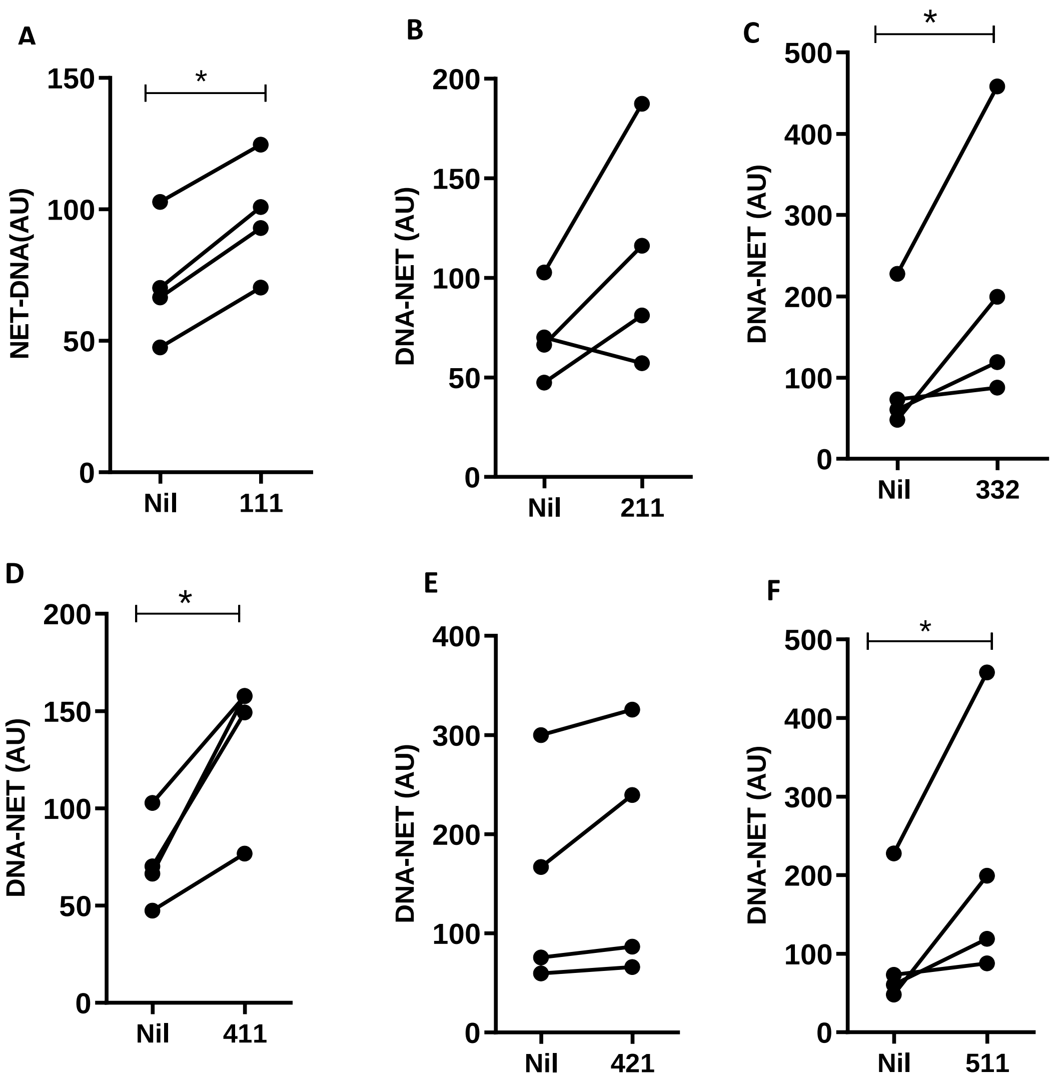

Figure S3. *Donor-to-donor variation in NET release induced by the indicated LM isoforms.* Neutrophils were incubated with the indicated concentrations of LM 111 (A), 211 (B), 332 (C), 411 (D), 421 (E) and 511 (F) for 90 min, and NETs in culture supernatants were quantified using PicoGreen. The data were normalized according to spontaneous release of DNA (Nil) and are shown as the mean ± SEM of 4 donors. * $p < 0.05$.

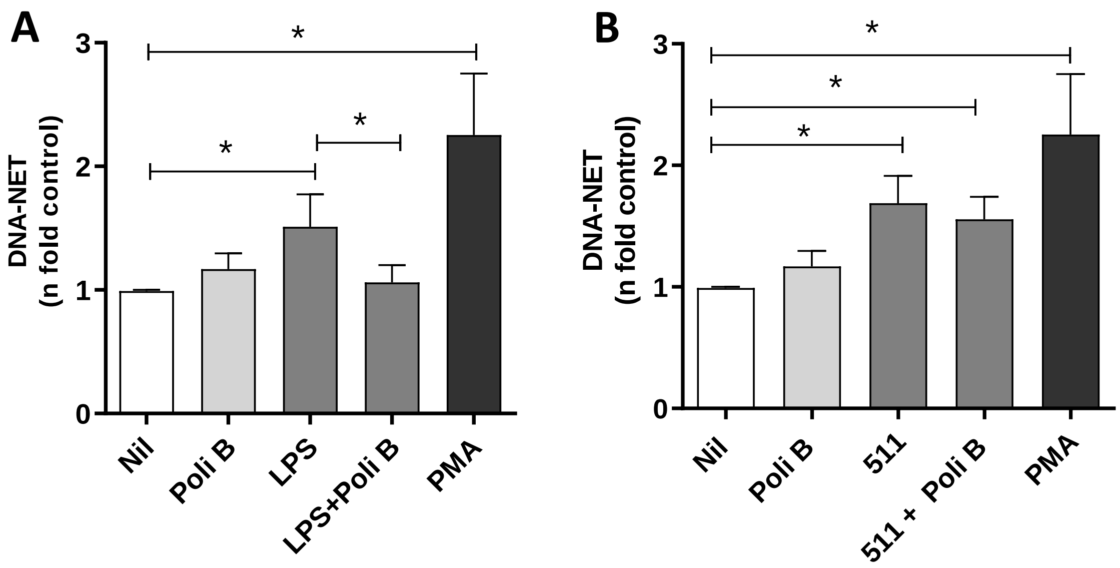

Figure S4*. NET induced by LM is not the result of LPS contamination*. Neutrophils (1x10^6) stimulated with LPS (2.5 µg/mL), LM-511 (5 µg/mL), were pretreated or not with Polymyxin B (20 µg/mL) for 30 min at 37°C, 5% CO_2. PMA (100 nM) was used as a positive control for NET induction. NETs were quantified in the culture supernatants with Picogreen. Data were normalized for spontaneous DNA release (Nil) and represented as the mean ± SEM from 8 donors in 4 independent experiments (A) and 6 donors in 3 independent experiments (B). * $p < 0.05$.

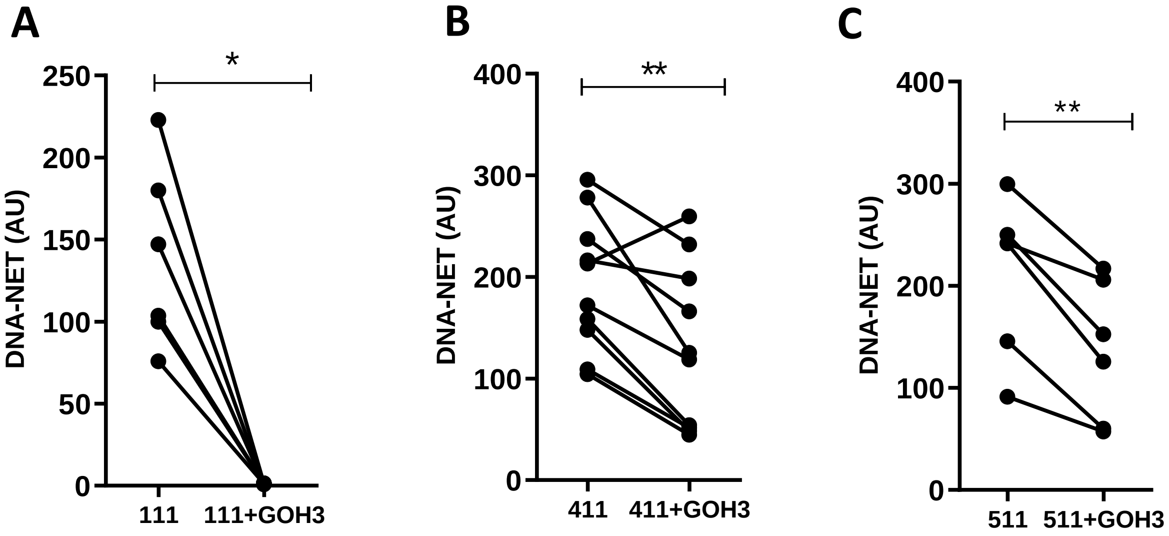

Figure S5. *Donor-to-donor variation in the NETs released and antibody-mediated inhibition of the α6 integrin chain*. Neutrophils were either treated or not (Nil) with anti-α6 integrin antibody (GoH3) for 20 min and then stimulated with laminin isoforms 111, 411 and 511 for 60 min. Following stimulation, quantification of DNA in the culture supernatants was performed using PicoGreen. The data are presented as the mean ± SEM of DNA-NET, and each dot represents a different donor. * $p < 0.03$; ** $p < 0.002$.

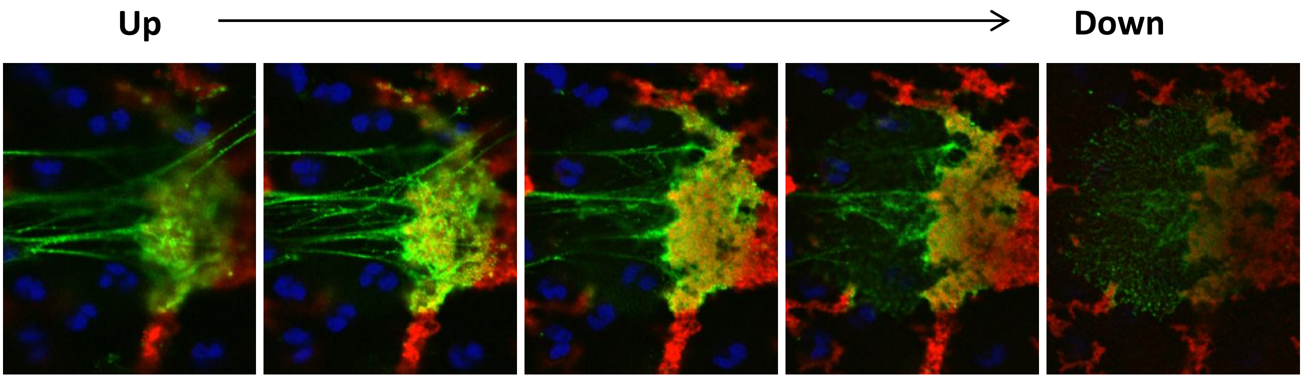

Figure S6*.* *NETs were entangled in PolyLM 111*. Neutrophils were incubated in polyLM 111 (50 μg/mL)-coated plates for 90 min, and cultures were fixed and stained with DAPI (blue) to detect DNA and with anti-elastase (green) and anti-α1 LM (red) antibodies. NETs (green) were observed on the LM surface layer (up) towards the bottom where the polyLM 111 (red) was adhered to the plate (down). The image shows an optically sectioned stack.

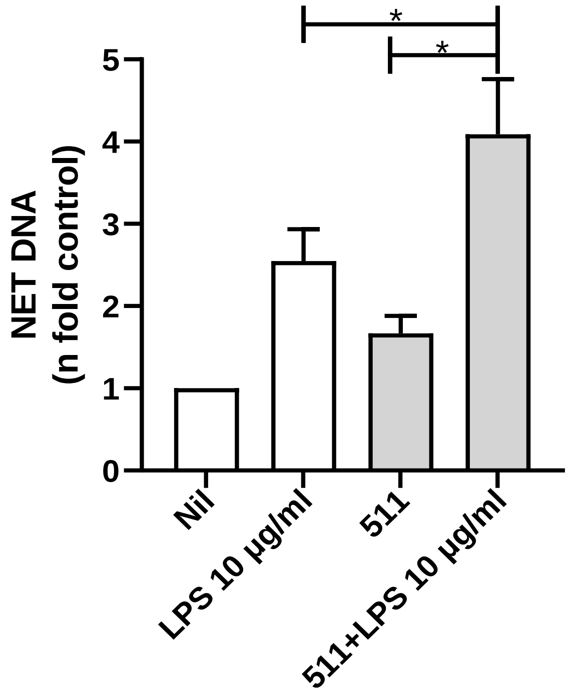

Figure S7. *LM-511 modulate NETs induced by E. coli LPS.* Neutrophils (10^5) seeded in 96-well plates were stimulated or not with 1 µg/mL LM 511 for 30 min and then further incubated in the presence or absence of 10 µg/mL delipidated *E. coli* LPS for 4 h. NETs in culture supernatants were quantified using PicoGreen. Data were normalized according to spontaneous release of DNA (Nil) and are represented as the mean ± SEM of 4 donors. * $p < 0.05$.
